# The Toll-Like Receptor Signaling Molecule *Myd88* Contributes to Pancreatic Beta-Cell Homeostasis in Response to Injury

**DOI:** 10.1371/journal.pone.0005063

**Published:** 2009-04-01

**Authors:** Paul L. Bollyky, Jeffrey B. Bice, Ian R. Sweet, Ben A. Falk, John A. Gebe, April E. Clark, Vivian H. Gersuk, Alan Aderem, Thomas R. Hawn, Gerald T. Nepom

**Affiliations:** 1 Benaroya Research Institute, Seattle, Washington, United States of America; 2 Institute for Systems Biology, Seattle, Washington, United States of America; 3 University of Washington School of Medicine, Seattle, Washington, United States of America; Instituto Oswaldo Cruz and FIOCRUZ, Brazil

## Abstract

Commensal flora and pathogenic microbes influence the incidence of diabetes in animal models yet little is known about the mechanistic basis of these interactions. We hypothesized that *Myd88*, an adaptor molecule in the Toll-like-receptor (TLR) pathway, regulates pancreatic β-cell function and homeostasis. We first examined β-cells histologically and found that *Myd88−/−* mice have smaller islets in comparison to C57Bl/6 controls. *Myd88−/−* mice were nonetheless normoglycemic both at rest and after an intra-peritoneal glucose tolerance test (IPGTT). In contrast, after low-dose streptozotocin (STZ) challenge, *Myd88−/−*mice had an abnormal IPGTT relative to WT controls. Furthermore, *Myd88*−/− mice suffer enhanced β-cell apoptosis and have enhanced hepatic damage with delayed recovery upon low-dose STZ treatment. Finally, we treated WT mice with broad-spectrum oral antibiotics to deplete their commensal flora. In WT mice, low dose oral lipopolysaccharide, but not lipotichoic acid or antibiotics alone, strongly promoted enhanced glycemic control. These data suggest that *Myd88* signaling and certain TLR ligands mediate a homeostatic effect on β-cells primarily in the setting of injury.

## Introduction

Toll-like receptors (TLRs) recognize structurally conserved microbial products and mediate the initiation of inflammatory and immune defense responses [1]. All TLRs, with the exception of TLR3, can signal via the adaptor *Myd88*, leading to activation of NF-κB as well as mitogen-activated protein (MAP) kinase pathways. These pathways have been shown to regulate immune response genes such as cytokines and chemokines that modulate both innate and adaptive immunity [2], [3].

In conjunction with their role in host defense, TLRs also play concomitant roles limiting tissue damage and promoting wound healing at sites of injury. This has been demonstrated for multiple tissue types including lung [4], liver [5], skin [6], and intestine [7]. Components of microbial pathogens interact with TLRs to promote production of several protective factors relevant to cytoprotection, tissue repair and angiogenesis. These include TGF-β1 [8], VEGF [9], KGF-1 [10] KGF-2 [11], HGF [12] and prostaglandins [13].

In the gut commensal flora-derived TLR ligands have been proposed to play a homeostatic role in steady state conditions [7]. TLRs contribute to liver regeneration [5] and are implicated in the pathogenesis of many chronic GI disorders, including celiac disease [14], inflammatory bowel disease [15], colon cancer [16] and non-alcoholic steatohepatitis [17]. Gut flora has also been suggested to contribute to obesity [18] and diabetes in murine models [19], [20].

Although human and mouse β-cells are known to express TLR2, 3, 4 and 9 [21], [22], little is known about their role in pancreas β-cell physiology and diabetes. Data are largely limited to autoimmune diabetes models. In this context various bacterial extracts and antibiotics [23], [24], [25], [26], [27], as well as viral mimetics [28], [29], [30], [31] have been found to impact the incidence of disease, presumably by impacting on β-cell specific immune tolerance.

A recent study examined the role of *Myd88* in the incidence of autoimmune diabetes in Non-Obese Diabetic (NOD) mice [32]. Those authors found that NOD *Myd88*−/− mice were protected from diabetes under sterile pathogen free (SPF) conditions but not under gnotobiotic conditions. Furthermore, the gnotobiotic-reared NOD *Myd88*−/− mice were protected from diabetes by exposure to SPF gut flora. These data cast new light on the observation that cleanliness in NOD mouse colonies directly relates to diabetes incidence and suggest that gut flora may act as an important tolerizing agent. However, these data are specific to autoimmunity mediated by cytotoxic lymphocytes and do not address any role that *Myd88*−/− and TLR might regularly play in β-cell development and injury responses under normal (e.g. non-autoimmune prone) circumstances.

In this work we have examined the role of TLRs in β-cell physiology in a non-autoimmune prone model, the C57Bl/6 mouse. We hypothesized that TLR-microbial interactions play a role in β-cell development and homeostasis and that these effects might be particularly relevant in settings of injury. This research highlights an important connection between microbial products and pancreas β-cell homeostasis in which beta cell resistance to damage-associated apoptosis is mediated by a TLR dependent pathway.

## Materials and Methods

### Mice

*Myd88−/−*, *TLR2−/−*, *Tlr4−/−*, mice (129SvJ×C57Bl/6 background) were derived as previously described [2] and backcrossed for six generations with C57Bl/6 mice. *Tlr5−/−* mice were derived and backcrossed to a C57Bl/6 background for eight generations as previously described [31]. *TLR3−/−* pancreases were provided by Dr. Richard Flavell at Yale University. Wild type C57Bl/6 mice were purchased from The Jackson Laboratory (Bar Harbor, ME). Mice were maintained in the specific pathogen-free AAALAC-accredited animal facility at the Benaroya Research Institute or at the Institute for Systems Biology and handled in accordance with institutional guidelines. All animal work was approved by the Benaroya Research Institute (BRI) Animal Care and Use Committee (ACUC), and the Institute for Systems Biology Animal Care and Use Committee.

### Reagents

Lipopolysaccharide (LPS) (*S. minnesota* R595)(cat. #L9764), lipoteichoic acid (LTA) from *S. aureus* (cat. #L2515), streptozotocin (cat. #S0130), vancomycin (cat. #861987), metronidazole (cat. #M3761), neomycin (cat. #N1876), ampicillin (cat. # A9518) were all obtained from Sigma-Aldrich (St. Louis, MO).

### Blood Glucose Measurement

Blood glucose was performed via saphenous vein bleeds using a One-Touch FastTake glucometer (LifeScan, Milpitas, CA). For intraperitoneal glucose tolerance tests (IPGTT), mice were fasted (given water only) for 8–12 h. Mice were then injected intraperitoneally with 1.0 mg/ml d-glucose (stock solution in PBS) at a dose of 1 g/kg body weight. Saphenous blood glucose readings were taken at 0, 30, 60 and 120 minutes post injection.

### Histologic Studies

Animals were sacrificed under anesthesia and pancreatic and liver tissues were frozen in Tissue-Tek OCT embedding media (Sakura Finetek, Torrance, CA). For staining of frozen tissues, 6 µm tissue slices were fixed in 10% neutral-buffered formalin, processed and embedded in paraffin. Staining was performed with a polyclonal guinea pig anti-pig insulin antibody (DAKO, Insulin A0564) at 1∶100 after 20 minute citrate heat induced epitope retrieval with an HRP labeled polymer visualization kit (DAKO, EnVision+ K4010) following the manufacturer recommended staining protocol. Slides were then counterstained with hematoxylin, cleared and mounted.

### Evaluation of β-cell volume and mass

Quantification of β-cell volume and mass were performed by point-counting morphometry of insulin-immunostained pancreatic sections, with minor adaptation to the method described by Weibel et. al. [33], and later applied by Bonner-Weir and colleagues [34], [35], [36]. The number of pancreases used per each breed of mouse as well as the ages and weights of the mice are detailed in Table 1. For each pancreas, an average of 6 independent sections at least 100 µm apart were analyzed. Each section was covered systematically using a digital camera (Diagnostic Instruments, Sterling Height, MI) attached to a Leica DM-IRB microscope (Leica Microsystems, Wetzlar, Germany). Approximately 200 non-overlapping fields from an average of 6 sections were evaluated per pancreas. Between 2–5 pancreases were evaluated for each breed of mouse in the study (Table 1). The ImageJ software computer program [37] was used to superimpose a 99 point grid over each image. The number of intercepts over β-cells and over non-β-cell pancreatic tissue was counted. The relative β-cell volume was calculated by dividing the intercepts over β-cells by the intercepts over total pancreatic tissue. This number was multiplied by the total mass of the pancreas to arrive at the total β-cell mass for the pancreas in question. Islet number was calculated on a per field basis using the same fields as were used to calculate relative β-cell volume. The definition of an islet was chosen as a cluster of insulin positive cells with a minimum of four visible nuclei

**Table 1:** Characteristics of mouse populations used for histological analysis of WT and TLR KO strains.

Strain	N	Age (Wks)	Wt (gms)	
			Mean	StDev
WT	5	10	21.6	1.1
MyD88−/−	4	10	21	0.8
TLR5−/−	3	10	23.5	1.7
TLR4−/−	3	10	21.6	0.6
TLR3−/−	2	10	ND	ND
TLR2−/−	3	8	17.6	3.1

Differences between strains were not significant. TLR3 mice were not weighed prior to their being sacrificed.

### Islet isolation and culture

Mice were anesthetized by intraperitoneal injection of sodium pentobarbital (35 mg/230 g body wt). Islets were prepared by injecting collagenase (10 ml of 0.23 mg/ml liberase; Roche Molecular Biochemicals, Indianapolis, IN) into the pancreatic duct and surgically removing the pancreas. The pancreases were placed into 15-ml conical tubes containing 5 ml of 0.23 mg/ml liberase and incubated at 37°C for 30 min. The digestate was filtered (400 µm stainless steel screen), rinsed (Hank's buffered salt solution), and purified in a gradient solution of Optiprep (Nycomed, Oslo, Norway). Islets were cultured for 18–24 h in RPMI media 1640 supplemented with 10% heat inactivated FBS before further experimentation. All procedures were approved by the University of Washington Internal Animal Care and Use Committee.

### Evaluation of Islet size

Individual islets were evaluated for size by dithizone staining [38] and their diameter was scored as <50, <100, <150, <200, <250, <300 using a grid marked at 50 uM increments. A total of 167 islets from 2 WT mice and 198 islets from 3 Myd88−/− mice were assessed in this manner.

### Islet response to glucose challenge

Islet function was assessed by monitoring the insulin secretory response of the purified islets according to the procedure described by the Edmonton group [39]. Islets were washed twice in 3 mM glucose Krebs-Ringer bicarbonate (KBR) solution (2.6 mmol/l CaCl_2_/2H_2_O, 1.2 mmol/l MgSO_4_/7H_2_O, 1.2 mmol/l KH_2_PO_4_, 4.9 mmol/l KCl, 98.5 mmol/l NaCl, and 25.9 mmol/l NaHCO_3_ (all from Sigma-Aldrich, St. Louis, MO) supplemented with 20 mmol/l HEPES/NaHEPES (Roche Molecular Biochemicals, Indianapolis, IN) and 0.1% BSA (Serological, Norcross, GA). 40 islets per condition were placed into a 96 well plate (10 islets per well in quadruplicate) containing 200 µl of either 3 mM glucose KRB or 20 mM glucose KRB and incubated for 2 hours at 37°C and 5% CO_2_. The supernatant was collected for insulin measurement. Insulin concentrations in these experiments were analyzed with a human insulin enzyme-linked immunosorbent assay (ELISA) kit (ALPCO Insulin ELISA kit, Windham, NH).

### Streptozotocin Treatment

Mice were treated with STZ at 40 mg/kg/day intraperitoneally for 4 consecutive days. STZ was administered within 10 min. of its dissolution. Mice in the untreated control group received citrate buffer (vehicle) alone. IPGTT was performed on fasted mice on days 0, 11, 18, and 25 following the initiation of the 4 day STZ treatment course. Animals were monitored for diabetes by obtaining periodic blood samples from the tail vein of non-fasted mice and glucose was measured by a blood glucose meter. Mice were considered diabetic when non-fasting blood glucose levels were >200 mg/dl for two consecutive days. Mice were weighed every other day for the determination of percent weight change and any mice with a >10% weight loss were sacrificed. Weight loss was calculated as: % weight change = (weight at day X-day 0/weight at day 0)×100. Animals were monitored clinically for rectal bleeding, diarrhea, and general signs of morbidity, including hunched posture and failure to groom. No mice became frankly diabetic, lost more than 10% of body weight, or died on this protocol. Mice were anesthetized and pancreases were removed at several time points following the start of STZ treatment including days 0, 7, 14, and 21.

### TUNEL Staining and apoptosis quantification

Frozen tissues were cut (5 µm), fixed (4% paraformaldehyde), permeabilized (Triton X-100), and stained using a TUNEL cell death detection kit (terminal-deoxynucleotidyl-transferase-mediated dUTP Nick End Labeling, Roche Diagnostics GmbH, Mannheim, Germany). Positive controls with DNase (Invitrogen, Carlsbad, CA) and negative controls with Day 0 mice and only TUNEL label solution were used. For the insulin staining, polyclonal guinea pig anti-insulin antibody (Abcam, Cambridge, MA) and AlexaFlour 568-conjugated goat anti-guinea pig fluorescent antibody (Molecular Probes, Eugene, OR) were applied in separate incubation steps for 1 hour at 37°C following TUNEL staining. Slides were coverslipped using VectaShield hard mount with DAPI (Vector Laboratories, Burlingame, CA). All pancreatic tissue slides were imaged using a Leica DM-IRB (Leica Microsystems, Wetzlar, Germany) fluorescent microscope equipped with a 4-Megapixel CCD SPOT digital camera (Diagnostic Instruments, Sterling Height, MI) and merged using SPOT advanced imaging software. Islet morphology was identified from the insulin and DAPI staining. Apoptotic cells within islets were counted from the merged images as DAPI nuclei with positive TUNEL staining. No insulin negative apoptotic cells were identified.

### Glucagon-like peptide 1 (GLP-1) measurement

Separate blood samples taken from fasting mice and then 90 minutes post-glucose challenge were added to ice-cooled tubes containing EDTA. DPP-IV inhibitor (Cat # DPP4)(Linco, St. Charles, MO) was added at 10 ul per ml of blood. The samples were centrifuged within 30 minutes of blood collection and plasma was removed and stored samples at −20°C. GLP-1 was subsequently measured using an ELISA kit (Linco, St. Charles, MO) according to the manufacturer's instructions.

### Depletion of gut commensal microflora and reconstitution of commensal-depleted animals with TLR ligands

WT animals were depleted of commensals using a 4-week oral antibiotic regimen. Mice were provided ampicillin (1 g/L), vancomycin (500 mg/L), neomycin sulfate (1 g/L), and metronidazole (1 g/L) in drinking water for four weeks prior to beginning STZ treatment and for the duration of the experiment. This protocol was based on previously described depletion protocols [7], [40]. For the determination of colonic microflora, fecal matter was removed from colons using sterile technique, placed in 15 ml conical tubes with thyoglycolate, and vortexed until homogenous. Contents were diluted and plated on universal and differential media for the growth of anaerobes and aerobes in the clinical microbiology lab in the Department of Laboratory Medicine of Virginia Mason Medical Center. After counting, colonies were picked and identified by biochemical analysis, morphologic appearance, and Gram staining. At week 3, drinking water was supplemented with 10 µg/µl, or 10 ng/µl of LPS or 100 ng/µl of *S. aureus* LTA and was continued in drinking water for the duration of the experiment. Eight age and gender matched mice were in each experimental arm. STZ treatment was initiated as described above.following depletion of intestinal flora.

### Statistical Analysis

Statistical analysis was performed using a Student's t test except for the comparison of islet size distributions, where a Kolmogorov-Smirnov (KS) test was used. P values<0.05 were considered significant. Error bars represent±SEM.

## Results

### *Myd88−/−* mice have reduced β-cell volume and islet size

To test the hypothesis that TLR signaling was critical for β-cell homeostasis, we first examined the impact of TLR signaling on pancreatic β-cell size and function in the absence of injury. We looked for phenotypic differences between WT mice and knock-out mice for *Myd88*, TLR2, TLR3, TLR4 and TLR5.

**Figure 1:**
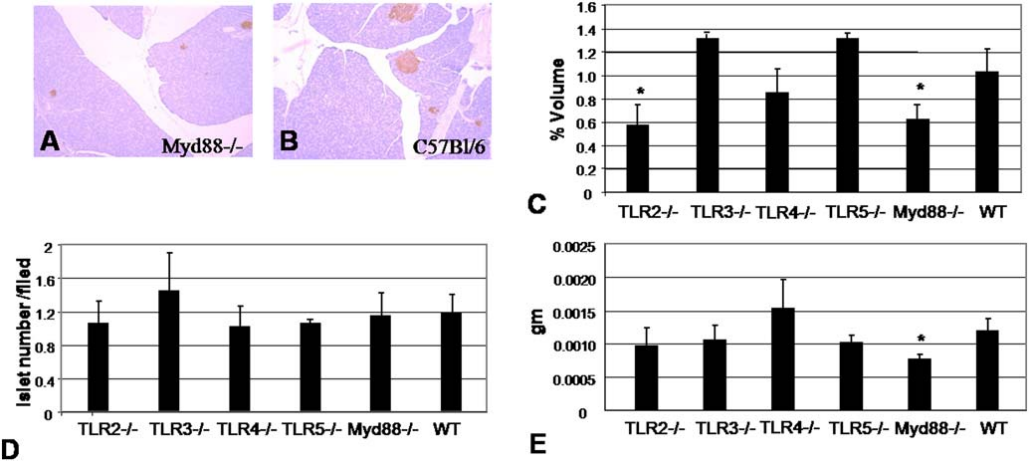
Histologic measurement of β-cell volume, mass and islet number for WT and TLR KO mice. A&B Representative histological sections from A) *Myd88*−/− and B) B6 mice. C. β-cell volume as a percentage of total pancreas volume. D. Islet cell number per histologic field examined. E. Total β-cell mass. The ages, body weights, and pancreatic weights of the mice used for these studies are shown in Table 1. * = p<0.05.

We focused initially on islet cell mass and volume because the cell mass of islets present in the pancreas, particularly that of β-cells, plays an essential role in determining the amount of insulin secreted. The numbers, ages and body weights of the mice used are shown in Table 1. Both TLR2−/− and *Myd88*−/− mice were found to have significantly smaller relative β-cell volume as a percentage of total pancreas volume (Figure 1C). However, the number of islets per field between the different mouse strains remained intact (Figure 1D), suggesting that *Myd88*−/− islets were smaller than WT islets. When total β-cell mass was calculated, only *Myd88*−/− mice had diminished β-cell mass (Figure 1E). Therefore, in our subsequent work, we elected to focus solely on the comparison between *Myd88*−/− and WT animals.

**Figure 2:**
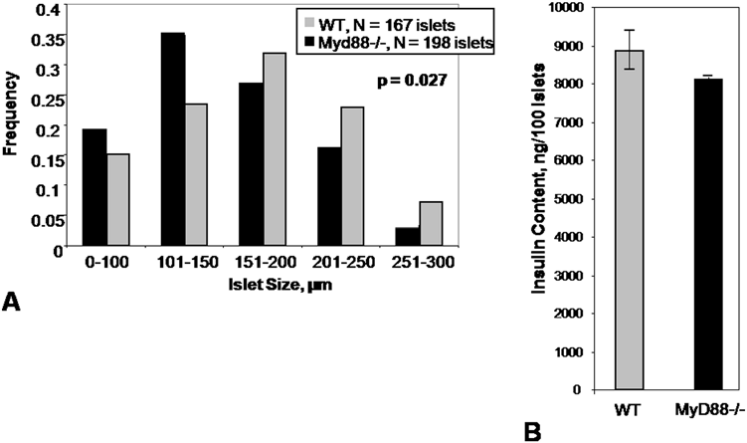
Size Distribution of Islets and pooled islet insulin content in WT versus *Myd88*−/− mice. A. Islets were harvested from *Myd88*−/− (3 mice) and WT (2 mice) animals and were graded by size. The two size distributions were significantly different (p = 0.027). B. Total insulin content of pooled WT versus *Myd88*−/− islets. Four groups of 10 size-matched islets were compared in each group. Differences between the groups were not significant.

This histologic finding of smaller *Myd88*−/− islets was born out by measurements of islet diameter *ex vivo* (Figure 2A). Pooled islets from *Myd88*−/− mice tended to have less insulin content, though this difference was not significant (Figure 2B). Together these data demonstrate a difference in β-cell volume and islet size between *Myd88*−/− and WT mouse strains and suggests *Myd88* may play a role in the regulation of β-cell mass.

### Uninjured *Myd88*−/− mice do not demonstrate impaired glycemic control

**Figure 3:**
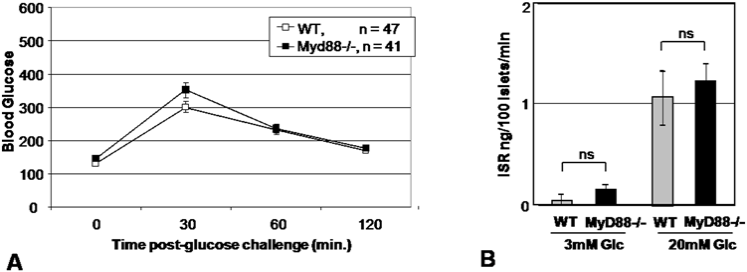
Response to glucose challenge *in vivo* and *ex vivo*. A. Pooled data are shown for IPGTT performed on 47 WT mice and 41 *Myd88*−/− mice. Only the 30 min time-point comparison was significant. All mice were between 10 and 20 weeks of age. The mean age was 15.5 (+/−2.1) weeks for WT mice and 16.0 (+/−3.8) weeks for *Myd88*−/− mice. The mean weight was 24.75 (+/−3.5) gms for WT mice and 23.4 (+/−3.6) gms for *Myd88*−/− mice. These differences in age and weight were not significant. 34% of the WT mice were female while 43% of the WT mice were female. IPGTT test results were not significantly different between the two genders. B. Insulin secretion in response to glucose challenge. Both total insulin content and insulin response to glucose challenge were determined in paired experiments using islets from the same pair of mice. 10 islets per well were assessed in quadruplicate per condition, per mouse. Data shown are representative of two experiments. There was no significant difference between the two strains of mice.

To examine the functional relevance of this difference in islet size, we measured fasting glucose as well as after an intraperitoneal glucose challenge (IPGTT) in *Myd88*−/− and WT mice. We found no difference between the two groups in fasting blood glucose or peak blood glycemia upon IPGTT challenge (Figure 3A). E*x vivo* evaluation of insulin production by WT and *Myd88*−/− islets matched for size likewise demonstrated no difference between the two strains upon stimulation with either low (3 mM) or high (20 mM) glucose concentrations (Figure 3B). We therefore concluded that while *Myd88*−/− animals have islets of smaller size this did not appear to have any functional consequence in otherwise healthy animals.

### *Myd88*−/− mice treated with STZ demonstrate impaired glycemic control and transaminitis

We next hypothesized that small decreases in β-cell mass mediate altered glucose regulation in settings of borderline glycemic control. We therefore designed experiments to stress animals into borderline glycemic control conditions to evaluate the functional relevance of the aforementioned decreases in islet cell size and mass. We administered 4 days of low-dose (40 mg/kg) STZ treatment to *Myd88*−/− and WT controls. While high dose STZ protocols (e.g. 200 mg/kg) reliably induce diabetes in C57Bl/6 mice in our experiment, our intention was to administer a sub-diabetogenic dose which might impact glycemic control of the two strains of mice differently. Following STZ treatment, we monitored the mice with weeklyat 0, 7, 14 and 24 days with IPGTT over a three week period.

**Figure 4:**
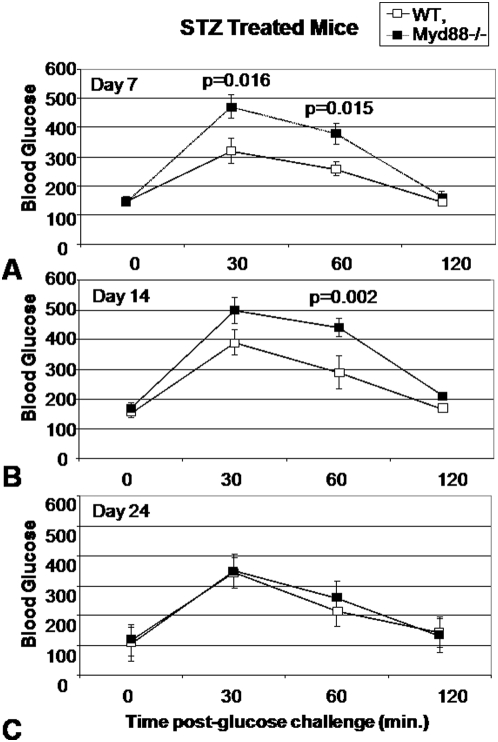
IPGTT for WT and *Myd88*−/− mice treated with STZ. Data shown are for 8 WT mice and 8 *Myd88*−/− mice who received low-dose STZ treatment for 4 days. IPGTT data are shown for this same group of mice 7 days (A), 14 days (B) and 24 days (C) following completion of the STZ treatment course. All mice were between 12 and 16 weeks of age and were evenly split between male and female genders. The mean weight was 21.3 (+/−2.3) gms for WT mice and 24.8 (+/−4.1) gms for *Myd88*−/− mice. This difference in weight was not significant. Data shown are representative of two experiments.

We found that *Myd88*−/− mice developed significant hyperglycemia relative to WT controls upon glucose challenge (Figure 4A–D). This was the case for both the 30 minute and 60 minute IPGTT time points on day 11 (7 days after the final dose of STZ)(Figure 4A) as well as for the 60 minute time point for day 18 (14 days after the last dose of STZ)(Figure 4B). The *Myd88*−/− mice did, however, recover normal glycemic control by day 28 (24 days after the final dose of STZ) (Figure 4C) consistent with healing. Of note, *Myd88*−/− mice did not reliably develop hyperglycemic responses to IPGTT upon treatment with 3 days of low-dose STZ treatment (data not shown), suggesting a threshold with the 4-day regimen.

**Figure 5:**
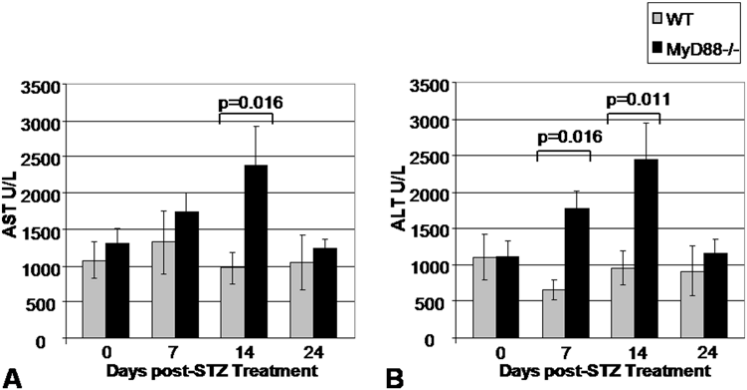
Transaminase Levels Following STZ Treatment. Data shown are for A. AST and B. ALT for 8 WT mice and 8 *Myd88*−/− mice who received low-dose STZ treatment for 4 days. The animals in this experiment were the same set of mice described in Figure 4 and data for the same time points 7, 14, and 24 days post completion of the low-dose STZ treatment course are shown.

In order to evaluate whether the enhanced responsiveness of *Myd88*−/− mice was a systemic feature of the *Myd88*−/− phenotype, we looked for evidence of heightened injury in another cell type susceptible to STZ damage—the hepatocyte. We evaluated aspartate transaminase (AST) and alanine transaminase (ALT) for the same time points as were evaluated with IPGTT. AST and ALT are liver transaminases which, when found in the peripheral circulation at elevated levels, are indicative of hepatocellular damage. We predicted that systemic administration of STZ would have a parallel impact on hepatocytes as on β-cells, as both cell types are sensitive to STZ. This was indeed the case for both AST and ALT (Figure 5A,B). These data are consistent with systemic hyper-responsiveness to STZ treatment in the absence of *Myd88*−/− signaling.

### *Myd88*−/− mice demonstrate enhanced β -cell apoptosis following low-dose STZ treatment

**Figure 6:**
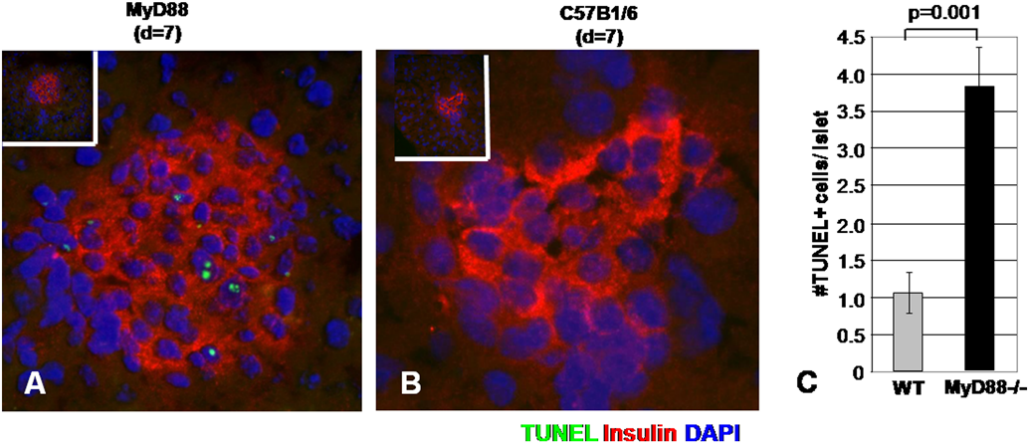
TUNEL staining of pancreatic sections from WT and *Myd88*−/− mice following treatment with STZ. Data are shown for sections taken from A. *Myd88*−/− and B. WT mice on day 7 following completion of a low-dose STZ treatment. TUNEL staining is shown in green, insulin staining is shown in red and DAPI is shown in blue. C. Pooled data for the number of TUNEL staining cells per islet in *Myd88*−/− versus WT mice.

The heightened cell damage observed in multiple tissue types in *Myd88*−/− animals raised the possibility that low-dose STZ treatment reflected an increased sensitivity to STZ-induced apoptosis. STZ is known to induce apoptotic cell death in hepatocytes as well as β-cells [41], [42]. We therefore compared TUNEL staining in pancreases from low-dose STZ-treated *Myd88*−/− mice and WT controls. This was done for 6 sections from two mice from each group taken before and after low-dose STZ treatment for 11 days. No TUNEL-positive islet cells were seen in the sections taken from mice prior to STZ treatment (data not shown). However following low-dose STZ treatment we observed an increase in TUNEL positive cells in islets from *Myd88*−/− mice (Figure 6A) but not WT controls (Figure 6B). This difference was significant (Figure 6E). While it is possible that *Myd88*−/− mice develop impaired glycemic control more readily upon STZ treatment because they start with diminished β cell mass, these data suggest their β-cells are also more likely to undergo apoptosis under these conditions.

### *Myd88*−/− mice do not have diminished levels of GLP-1

To rule out a *Myd88*-dependent effect mediated by glucagon-like peptide-1 (GLP-1), a soluble factor known to promote β-cell mass and insulin gene expression [43], [44], we analyzed GLP-1 concentrations in serum from mice both fasting (time = 0′) and following IP glucose challenge (time = 90′) (Figure S1). We did not find any differences in GLP-1 content between the two strains, suggesting that the findings reported here are not due to differences in GLP-1 expression.

### Low-dose oral LPS promotes glycemic control in animals with impaired commensal flora

**Figure 7:**
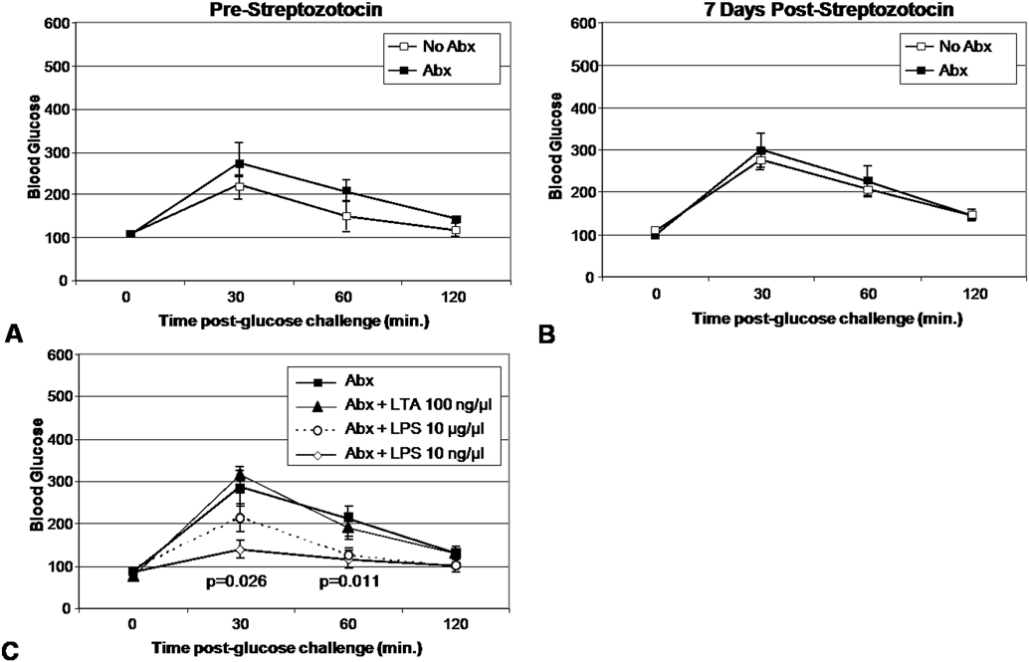
Chronic broad-spectrum oral antibiotic treatment does not confer enhanced susceptibility to low-dose STZ treatment. Mice received a cocktail of oral antibiotics (vancomycin 500 mg/L, metronidazole 1 g/L, neomycin 1 g/L, and ampicillin 1 g/L in drinking water) for 4 weeks prior to STZ administration. IPGTT were performed prior to (A) and 7 days subsequent to (B) completion of a 4 day course of low dose STZ treatment. 8 mice were in each experimental arm. C.IPGTT results for mice who received daily supplementation of their drinking water with LTA at 100 ng/µl, LPS at 10 ug/µl, or LPS at 10 ng/µl in addition to supplementation with broad-spectrum antibiotics. The mice shown did not receive STZ. 8 mice were in each experimental arm. These experiments were performed twice with similar results.

Based on the above data we hypothesized that commensal flora provide anti-apoptotic signals through *Myd88*−/−. This hypothesis predicts that denuding WT mice of their intestinal flora enhances sensitivity to low-dose STZ treatment and recapitulates the *Myd88*−/− phenotype. Therefore we administered a cocktail of broad spectrum antibiotics to WT animals for four weeks [7]. We found that these mice were normoglycemic before and after low-dose STZ treatment in comparison to mice which did not receive antibiotics (Figure 7A–B). Interestingly, we found that four weeks after the initiation of antibiotics we were still able to culture limited numbers of viable fecal bacteria. We then attempted to rescue TLR signaling in ABX treated mice by supplementing drinking water with LPS or LTA. We found that LPS supplementation of drinking water in these mice promoted tight glycemic control (Figure 7C). This effect was significant in the group of mice which received 10 ng/µl LPS but not in the groups recieving 10 µg/µl nor the group which received LTA. None of the mice in these experiments, either those which received antibiotics nor those which received antibiotics plus TLR ligands, demonstrated a significant change in their glycemic control from baseline upon IPGTT following low-dose STZ treatment (data not shown).

## Discussion

The first noteworthy finding of our manuscript is that TLRs and the signaling molecule *Myd88* in particular may contribute to the regulation of islet mass. The histologic measurements reported here point to a loss of approximately 40% more islet volume in *Myd88*−/− animals as compared to WT controls on the same C57Bl/6 background. As TLR represent a major molecular interface between hosts and microbial flora, this finding may help explain an isolated report of diminished islet mass in gnotobiotic rats [45]. As no specific individual TLR was associated here individually with diminished islet mass, it may be that a combination of TLR make a contribution towards maintaining islet homeostasis in WT animals.

The second finding of this work is that *Myd88*−/− mice were more likely than WT controls to develop hyperglycemia upon β-cell insult. *Myd88*−/− mice became relatively hyperglycemic upon IPGTT following treatment with a low-dose STZ regimen. This is in contrast to the intact glycemic control seen here in healthy, uninjured *Myd88*−/− mice upon fasting IPGTT and evaluation of their islets *ex vivo*. It may be that *Myd88*−/− animals maintain adequate functional reserve to maintain glycemic control under normal circumstances and their reduced islet mass only become functionally relevant upon injury. This is consistent with models of diabetes in which the large majority of β-cell mass must be lost before hyperglycemia is observed.

The third notable finding of this work is that *Myd88*−/− mice also have an increased rate of apoptosis upon low-dose STZ treatment,as *Myd88*−/− mice had greater numbers of apoptotic TUNEL positive cells per islet than WT controls. Both enhanced islet injury upon STZ treatment and diminished islet mass may contribute to the hyperglycemia seen here in *Myd88*−/− mice. The conclusion that *Myd88−/−* suffer greater injury upon STZ treatment is buttressed by the fact that they also displayed transaminitis indicative of hepatocyte damage. *Myd88* mediated activation of NF-kB is known to promote cell survival via production of anti-apoptotic Bcl-2 family molecules [46], [47], [48], [49]. However, *Myd88* also binds the Fas-associated Death Domain (FADD) and promotes induction of apoptosis via a pathway involving caspase 8 [50]. In the context of the pancreas, TLR4−/− mice were found to have reduced apoptosis in a surgical model of pancreatitis [51]. Whether *Myd88* signaling contributes toward anti- or pro-apoptotic pathways is dependant on the particular TLR involved and other contextual cues [52]. While TLR have been found to contribute to the maintenance of tissue integrity at multiple sites of host-microbial interface [4], [5], [6], [7], these data extend this model to two tissue types not always considered to be part of that interface, the pancreatic islet and liver.

The enhanced sensitivity to low-dose STZ was not recapitulated in WT mice denuded of intestinal flora upon the long-term administration of broad-spectrum antibiotics. Since this treatment did not render the intestines of these mice entirely sterile, it may be that even small quantities of intestinal bacteria are adequate. Alternatively, endogenous TLR ligands might suffice to maintain islet homeostasis upon STZ treatment in the setting of competent *Myd88* signaling.

Our data do not contradict the recent finding that the absence of *Myd88* leads to changes in the penetrance of diabetes in NOD mice raised in either SPF or gnotobiotic conditions. In our work diabetes is induced by a toxin whereas diabetes in NOD mice is mediated by an autoimmune phenomenon. Together these data emphasize that effects of different TLR ligands and gut microbiota on diabetes are likely to be complex, multifactorial and dependent on the genetic underpinnings of the diabetes in question.

Our data also do not directly contradict the recent report that *Myd88−/−* mice developed an increased incidence of autoimmune diabetes relative to WT controls one to two months after low-dose STZ treatment. Notably those authors did not find relative hyperglycemia in *Myd88−/−* mice in the weeks immediately following low-dose STZ treatment but they measured blood glucose in fasting mice rather then upon glucose challenge via IPGTT as we have done here.

We were intrigued to find that low-dose oral LPS has a salutary effect on glycemic control. These data are consistent with reports that endogenous gram negative bacteria tonically prime insulin production in rats [53] and consistent with data that treatment of islets *ex vivo* with LPS promotes β-cell insulin production [21]. In that study the tonic effects of LPS on insulin secretion were dose dependant, with the lower concentrations being more beneficial. These effects were observed at LPS concentrations of 1–100 ng/ml, which is similar to that used to stimulate macrophages. At higher concentrations of LPS (e.g. 5–10 µg/ml) cytotoxic effect on islets have been observed [54], [55] and *in vivo* are associated with sepsis-induced hypoglycemia [56]. In sum, low-dose LPS appears to promotes insulin production and release while high dose LPS leads to stress-related responses. The dose-dependent nature of LPS effects on β-cells may be clinically relevant during acute endotoxemic events and in settings of inflammation. Chronic inflammation and basal endotoxemia have been correlated with obesity and type 2 diabetes in humans and rats [19], [57]. Dose-dependant effects of endotoxin on β-cells may also be relevant to the viability and function of transplanted islets. In the most commonly followed transplant protocol, endotoxins are infused into the portal circulation of recipients [58], where levels of endotoxin have been shown to be higher relative to peripheral circulation [59].

We interpret the effects observed upon low-dose oral LPS treatment as functionally distinct from the role described here for *Myd88* signaling in the setting of β-cell injury. The salutary effect of oral LPS was apparent irrespective of STZ treatment and in the absence of injury. *Myd88*−/− mice, conversely, were phenotypically unremarkable until they received STZ. While these findings appear to describe distinct physiologic processes, they may be mechanistically related. Such overlap between pathways involved in β-cell survival and β-cell autoimmunity may account for the complex and sometimes contradictory relationship between infection and type 1 diabetes. Together these data suggest that TLRs and their ligands may play important roles in β-cell physiology in both health and injury. They point towards an additional frontier in diabetes research, the role of microbial products in the regulation of glycemic control.

## Supporting Information

Figure S1 Myd88−/− mice do not have diminished levels of GLP-1. Serum was taken from mice both fasting (time = 0′) and following IP glucose challenge (time = 90′) and subsequently analyzed for GLP-1 content. Data shown are for 12 WT mice and 8 Myd88−/− mice.
(0.05 MB TIF)
