# Supplementary figures and images for: The Toll-Like Receptor Signaling Molecule Myd88 Contributes to Pancreatic Beta-Cell Homeostasis in Response to Injury
Source: PLoS One. 2009 Apr 1;4(4):e5063. doi: 10.1371/journal.pone.0005063 (PMC2666970; doi:10.1371/journal.pone.0005063)

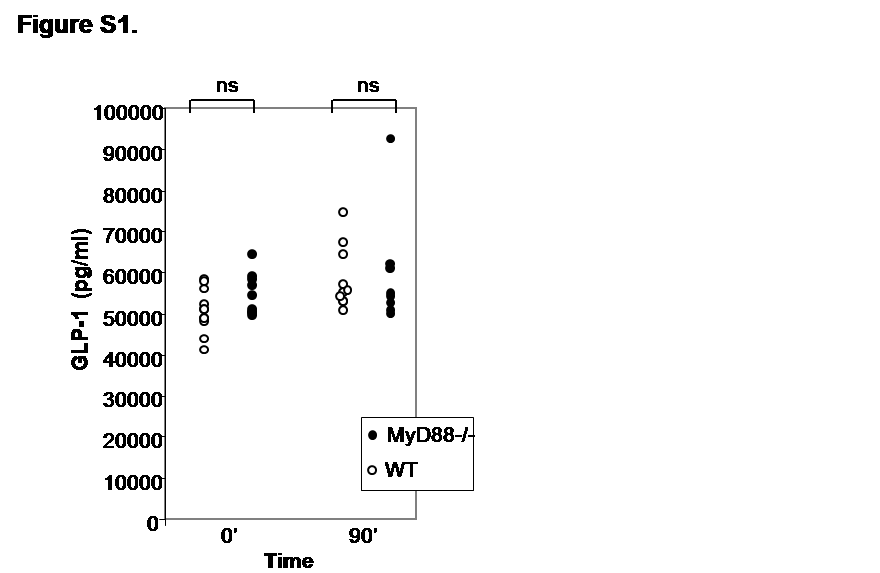

Supplement: Figure S1 — Myd88−/− mice do not have diminished levels of GLP-1. Serum was taken from mice both fasting (time = 0′) and following IP glucose challenge (time = 90′) and subsequently analyzed for GLP-1 content. Data shown are for 12 WT mice and 8 Myd88−/− mice. (0.05 MB TIF) [file pone.0005063.s001.tif]
